# Alcoholism and Alternative Splicing of Candidate Genes

**DOI:** 10.3390/ijerph7041448

**Published:** 2010-03-30

**Authors:** Toshikazu Sasabe, Shoichi Ishiura

**Affiliations:** Department of Life Sciences, Graduate School of Arts and Sciences, the University of Tokyo, 3-8-1, Komaba, Meguro-ku, Tokyo, 153-8902, Japan; E-Mail: cc087707@mail.ecc.u-tokyo.ac.jp

**Keywords:** alcoholism, alternative splicing, dopamine, NMDA, GABA, voltage-gated calcium channel, neurexin

## Abstract

Gene expression studies have shown that expression patterns of several genes have changed during the development of alcoholism. Gene expression is regulated not only at the level of transcription but also through alternative splicing of pre-mRNA. In this review, we discuss some of the evidence suggesting that alternative splicing of candidate genes such as DRD2 (encoding dopamine D2 receptor) may form the basis of the mechanisms underlying the pathophysiology of alcoholism. These reports suggest that aberrant expression of splice variants affects alcohol sensitivities, and alcohol consumption also regulates alternative splicing. Thus, investigations of alternative splicing are essential for understanding the molecular events underlying the development of alcoholism.

## 1. Introduction

Alternative pre-mRNA splicing makes a large contribution to proteomic diversity. In alternative splicing, various potential splice sites of the pre-mRNA from a single gene are used in various combinations that lead to the translation of several functionally distinct protein isoforms from several different mRNA species. In the brain, the regulation of splice variants modulates protein functions, which can ultimately affect behavior such as alcohol dependence. Alcohol dependence (AD) is a common, chronic and relapsing disorder with an estimated heritability of 40−60% [1]. Family and twin studies have shown that genetic factors contribute to the risk of AD and genetic mapping studies have identified numerous genes associated with this risk [2–5]. Most of these genes affect ethanol metabolism, ethanol preference, or diverse brain systems, such as the reward system [6,7].

Alterations in expression have been shown to be involved in producing neuroadaptative changes following chronic ethanol consumption [8,9]. A key question in AD is the transition from controlled to compulsive drinking, and development of dependence may be related not only to gene expression modulated through transcriptional regulation but also through alternative splicing of genes, which may produce functionally distinct isoforms.

In this review, we describe several examples of alternative splicing which may affect ethanol preference and consumption. We propose that alternative spliced forms may be important to the development of alcoholism.

## 2. Ethanol-Associated Genes

### 2.1. The D2 Dopamine Receptor

The mesolimbic dopamine system plays an important role in the rewarding and reinforcing effect of drugs on the brain [10,11]. It is thought to play a similar role in the rewarding effect of ethanol, as ethanol administration induces the release of dopamine in the nucleus accumbens [12].

Of the five dopamine receptor subtypes, the D2 receptor subtype (DRD2) has been extensively studied in alcoholism [13–23]. In rats, a decreased DRD2 density is associated with a preference for ethanol [14], and transient overexpression in the nucleus accumbens attenuated ethanol preference [15]. In humans, positron emission tomography studies have revealed decreased DRD2 availability in the striatum of alcoholics [16,17], and support a hypothesis that a high DRD2 level in the striatum is a protective factor against alcoholism [18]. Moreover, polymorphisms of the DRD2 gene are linked with an altered risk of alcoholism [19–23].

Alternative splicing generates two DRD2 isoforms, D2S and D2L. These isoforms differ in that D2L has a sixth exon, which encodes its third cytoplasmic region [24]. Although the two isoforms are co-expressed, D2S is preferentially expressed in dopaminergic neurons in presynaptic regions, such as the substantia nigra and the hypothalamus, and D2L predominates in postsynaptic regions, such as the striatum and the pituitary gland [25–27]. In recombinant cell lines and mouse brains, D2L has a lower affinity for dopamine than D2S [24,28,29]. DRD2 is coupled with G_αi_, an adenylyl cyclase-inhibiting G protein, and because G_αi_ binding is mediated by the third cytoplasmic loop of DRD2, D2S and D2L have different specificities for the subtypes of G_αi_ [30–32].

The D2S and D2L isoforms also differ in their effects on downstream protein phosphorylation. D2S has been suggested to decrease the phosphorylation of tyrosine hydroxylase, and D2L has been suggested to increase the dopamine D1 receptor (DRD1)-induced phosphorylation of DARPP-32 (dopamine- and cAMP-regulated phosphoprotein of 32 kDa) [33]. In addition, D2S and D2L have been reported to differ in the levels of their accumulation in the endoplasmic reticulum (ER) [34] and in the regulation of their sequestration by G protein-coupled receptor kinases and β-arrestins [35]. Furthermore, although both D2L and D2S participate in the presynaptic inhibition of γ-aminobutyric acid (GABA) release in the striatum, the presynaptic inhibition of glutamate release is preferentially modulated by D2S [36]. D2L-deficient mice showed distinct pharmacological behavior from DRD2-deficient mice and wild-type mice, suggesting that the D2L splice variant has different functions from D2S *in vivo* [27,29,37,38].

Ethanol affects the alternative splicing of DRD2 pre-mRNA [39]. Ethanol administration to rats has been reported to increase the D2L/D2S receptor ratio in the pituitary gland, and this effect was reproduced in primary cultures of pituitary cells treated with ethanol. We separately confirmed this effect of ethanol on DRD2 splicing in the human neuroblastoma cell line SH-SY5Y (unpublished data). In pituitary cells, ethanol not only alters DRD2 splicing, but also diminishes the inhibition of prolactin secretion by the DRD2-specific analog bromocriptine [39]. These facts suggest that ethanol suppresses dopamine-induced responses by altering DRD2 splicing. Allelic expression analysis in human postmortem brain tissues and functional magnetic resonance imaging measurements in healthy humans have shown that T carriers of single-nucleotide polymorphisms (SNPs) rs2283265 and rs1076560, both of which flank DRD2 exon 6, express relatively fewer D2S receptors and exhibit worse performance during working tasks [40]. We have demonstrated that T carriers of SNP rs1076560 have a higher alcoholism risk [22].

These findings suggest that individuals possessing fewer D2S receptors, such as T carriers, have less intense responses to dopamine (like ethanol-preferring rats) and therefore prefer to drink alcohol. Furthermore, this tendency is enhanced through a positive feedback loop in which ethanol administration decreases the number of D2S receptors.

### 2.2. The N-Methyl-d-aspartate (NMDA) Receptor NR1 Subunit

The NMDA (N-methyl-D-aspartate) receptor, an ionotropic glutamate receptor in the central nervous system, is a pivotal target of ethanol [41]. Ethanol attenuates NMDA receptor function in a dose-related fashion *in vitro* [42,43]. The NMDA receptor is a heteromeric protein composed of at least one NR1 subunit and at least one NR2 subunit. Ethanol appears to bind to the NR1 subunit in a hydrophobic pocket associated with the third transmembrane domain [44]. Ligand binding to the NMDA receptor is up-regulated in rodents chronically treated with ethanol [43,45–48] and in postmortem cortical tissue from alcoholic patients [49,50]. This up-regulation of the NMDA receptor reduces ethanol sensitivity and therefore contributes to ethanol tolerance [51].

The NMDA receptor is a cation channel with high Ca^2+^ permeability. The NR1 subunit is essential for receptor–channel function, and the NR2 subunit modulates the properties of the channel [52]. The recently identified NMDA subunit NR3 decreases NMDA-induced current and Ca^2+^ permeability [53,54].

The NR1 subunit has eight isoforms generated by alternative splicing of exons 5, 21, and 22. The NR1-a variant lacks exon 5, which encodes the N1 splice cassette that lies in the extracellular N-terminal domain of the NR1 subunit, and NR1-b includes exon 5. Exons 21 and 22 encode the C-terminal splice cassettes C1 and C2, respectively, and are part of the intracellular domain of the NR1 subunit. NR1 splice variants lacking exon 22 contain a C2’ cassette as an additional cassette at their C-terminal end (see Figure 1 in reference [55]). These splice variants vary considerably in their properties and are differentially localized in adult and developing animals [56,57].

These alternative cassettes modulate the properties of the NMDA receptor. The presence of the N1 cassette, which forms a surface loop with structural similarities to polyamines, decreases the affinities of the receptor for NMDA and glutamate [58]. It potentiates the receptor function by relieving the tonic proton inhibition [59] and voltage-independent zinc inhibition [60]. It also accelerates the deactivation time course of NMDA responses when NR1 is co-expressed with the NR2B (but not NR2A) subunit [61,62]. The first C1 cassette includes a calmodulin-binding site, and binding of calmodulin to NR1 inhibits NMDA channel activity [63–65]. Although the C1 cassette also has protein kinase C (PKC) phosphorylation sites, and phosphorylation affects the subcellular distribution of NR1 [66], PKC potentiation of the NMDA receptor probably does not occur through direct phosphorylation of the receptor but, rather, through phosphorylation of receptor-associated proteins [67,68].

Interestingly, the NR1 isoforms have differential sensitivities to ethanol-induced inhibition. When expressed singly in *Xenopus* oocytes, the sensitivity of the NR1-1b, NR1-2b, NR1-1a, and NR1-2a isoforms to ethanol follows the order NR1-1b > NR1-2b > NR1-1a > NR1-2a [69]. In contrast, when all eight NR1 isoforms are co-expressed in various combinations with one of the four NR2 subtypes in human embryonic kidney 293 cells, the sensitivity depends on the combination. For example, the NR1-3b/NR2C, NR1-3b/NR2D, and NR1-4b/NR2C pairs are most weakly inhibited by ethanol (approximately 15% inhibition), and the NR1-2b/NR2C pair is most strongly inhibited by ethanol (>50% inhibition) [70].

Several reports have described the modulation of expression of specific NR1 isoforms by ethanol [54,57,71–77]. Some of the reported findings appear to be inconsistent. For example, although mRNA ratio of NR1 splice variants containing exon 5 to those lacking exon 5 (+E5/–E5) decreases in the cortex of rats exposed to ethanol vapor [72] and in mouse fetal cortical neurons treated with ethanol [73], it increases in the striatum of rats chronically treated with ethanol solution [77]. Raeder *et al*. [77] postulated that this inconsistency arises from differences in the dose, duration of consumption, and route of administration of alcohol. In addition, the effects of ethanol on the expression of NR1 isoforms might differ in each brain region.

Thus, despite this inconsistency, ethanol consumption may increase NR1 isoforms that are weakly inhibited by ethanol. Together with up-regulation of the NMDA receptors [37,39–44], this regulation of splice variants should contribute to the reduction of ethanol sensitivity and the induction of ethanol tolerance.

### 2.3. The GABA_A_ Receptor γ2 Subunit

The ionotropic GABA_A_ receptor, a GABA-gated chloride channel receptor, is involved in the diverse effects of ethanol on the central nervous system [78]. The ionotropic GABA_A_ receptor and the metabotropic GABA_B_ receptor mediate the inhibition of neuronal excitability by GABA. The majority of fast synaptic inhibition is mediated through the GABA_A_ receptor. Acute ethanol administration enhances GABA_A_ receptor activity at intoxicating concentrations, whereas chronic ethanol administration decreases GABAergic function [79–81]. Together with up-regulation of the NMDA receptor, this down-regulation of the GABA_A_ receptor contributes to ethanol tolerance, dependence, and withdrawal hyperexcitability. Ethanol directly binds to a pocket located between transmembrane domains of the GABA_A_ receptor, inducing conformational changes [82–84]. Dopaminergic neurons in the midbrain are activated not only directly through ethanol exposure, but also indirectly through the down-regulation of GABAergic inhibitory transmission to these neurons [85–87].

The GABA_A_ receptor is a heteromer of five subunits. Almost all GABA_A_ receptors consist of two α, two β, and one γ subunit; the most frequent combinations are α1β2γ2, α2β3γ2, and α3β3γ2, which comprise approximately 60, 15, and 10% of GABA_A_ receptor proteins, respectively [88]. Genes for these subunits form clusters on several chromosomal regions; 4p13–q11 (α2, α4, β1, γ1), 5q34–q35 (α1, α6, β2, γ2), 15q11–q13 (α5, β3, γ3), and Xq28 (α3, β4, ɛ1). Mice with knockouts of these genes generally do not exhibit alcohol-preferring behaviors [78]. Moreover, agonists of the GABA_A_ receptor enhance alcohol-drinking behavior, and antagonists inhibit it [89–91]. These results illustrate the importance of the GABA_A_ receptor in alcohol consumption.

The GABA_A_ receptor α2 subunit has four major isoforms differing in their combinations of two alternative exons (exon 1A *versus* exon 1B and exon 9A *versus* exon 10) and two minor isoforms lacking exon 4 or exon 8 [92]. Two haplotypes of the α2 subunit gene (*GABRA2*) have been reported to be significant risk factors for AD, although this conclusion is inconsistent with several other studies [93]. Some non-coding variations in the *GABRA2* gene have been found to be associated with AD [94]. Because several SNPs are located in exon 8, exon 9, or in the introns flanking these exons, one can speculate that these SNPs affect the alternative splicing of these exons.

The γ2 subunit has two splice variants, one long (γ2L) and one short (γ2S). The γ2L variant differs from the γ2S variant in that it has an eight-amino-acid insert in the intracellular loop between transmembrane domains three and four. Intriguingly, in rats, chronic intermittent ethanol administration decreases the hippocampal ratio of γ2L/γ2S [95]. The γ2L insert modulates GABA_A_ receptor function; it contains a serine residue that is phosphorylated by PKC, reducing the amplitudes of GABA-activated currents [96]. In addition, when γ2L is co-expressed with α and β subunits in *Xenopus* oocytes, only the γ2L subunit is sensitive to the reinforcing effect of ethanol [97]. It also exhibits accelerated deactivation in human embryonic kidney 293 cells [98]. Knockout mice lacking this insert (γ2L-KO) appear to be unaffected in their electrophysiological and behavioral response to ethanol [99], contrary to the different effects of the isoforms mentioned above. On the other hand, γ2L accumulates at inhibitory postsynaptic sites more effectively than γ2S, and this accumulation is facilitated by PKC phosphorylation of the γ2L insert [100]. Meier and Grantyn argue that γ2S functions sufficiently in γ2L-KO mice only because it does not have to compete with γ2L for gephyrin binding sites and that it actually differs from γ2L in its properties [100]. Moreover, expression of the γ2L and α1 subunits increases during development; they are the predominant isoforms in mature GABAergic synapses [101]. Thus, a reduced γ2L/γ2S ratio probably interferes with the maturation of inhibitory synapses and may indicate a higher risk of alcoholism.

### 2.4. N-Type and L-type Voltage-gated Ca^2+^ Channels

Opening of voltage-gated Ca^2+^ channels depends on the membrane potential. The resulting influx of Ca^2+^ into the cell triggers various events including neurotransmitter release. Ca^2+^ channels are composed of at least three subunits (α_1_, α_2_δ, and β). The α_1_ subunit is critical for Ca^2+^ channel functions, and its various subtypes confer functional diversity.

The N-type Ca^2+^ channel is involved in the molecular and behavioral effects of ethanol. Acute ethanol exposure inhibits the activity of the N-type channel in PC12 cells [102], whereas chronic ethanol exposure increases the density of N-type channels and therefore increases depolarization-evoked Ca^2+^ influx [103]. In addition, mice lacking N-type channels exhibit reduced voluntary ethanol consumption and preference [104]. Treatment with the mixed N-type and T-type channel antagonist NP078585 has a phenotypic effect similar to that of ethanol [105].

The α_1_ subunit of the N-type channel is α_1_2.2, which has three alternative exons (exon 18a, exon 24a, and exon 31a). The splice variants of these exons exhibit functionally diverse Ca^2+^ signaling in neurons. Exon 31a encodes two amino acids in the IVS3-IVS4 linker, altering the rate of channel opening and the voltage dependence of channel activation [106,107]. Chronic ethanol exposure decreases expression of the variant lacking exon 31a and increases the N-type current [108], suggesting that ethanol facilitates the functions of N-type Ca^2+^ channels by altering the composition of splice variants.

The L-type channel is also involved in ethanol consumption. Acute ethanol exposure inhibits the L-type channel, whereas chronic ethanol exposure induces a rise in the voltage-gated Ca^2+^ current, an effect thought to reflect up-regulation of the L-type channel [109,110]. In addition, L-type channel antagonists decrease ethanol withdrawal symptoms and ethanol self-administration in rodents [111,112].

The α_1C_ subunit of the L-type channel has two splice variants, α_1C-1_ and α_1C-2_. These variants differ in that α_1C-1_ has three additional amino acid residues between domains II and III and 28 additional residues in the S3 segment in domain IV. They are differentially expressed in rat brain [113], but little is known about their differences in function. Ethanol treatment in PC12 cells has been reported to increase the level of α_1C_ protein in a PKCδ activity-dependent manner, whereas it increases the mRNA level of α_1C-1_, but not α_1C-2_, independent of PKCδ [114]. These results indicate that ethanol up-regulates the L-type channel by increasing expression of the α_1C-1_ splice variant, in addition to PKCδ-dependent and post-transcriptional regulation.

Ethanol consumption appears to alter the ratios of splice variants, thereby up-regulating N- and L-type calcium channels. This up-regulation appears to induce the neuronal hyperexcitability observed during ethanol withdrawal.

### 2.5. Large-Conductance Calcium- and Voltage-Activated Potassium Channels (BK)

BK channels are large-conductance calcium- and voltage-activated potassium channels. K^+^ currents carried by BK channels participate in repolarization and hyperpolarization of action potentials, setting neuron firing properties [115]. BK channels consist of α core and β auxiliary subunits. The α core subunit forms a ion-selective pore, a voltage-sensing module, and a Ca^2+^-sensing module important for activation of the channel [116].

The activity of BK channels is modified by clinical doses of ethanol. Ethanol exposure potentiates BK activity in cell-free membranes and a cultured explant from rat posterior pituitary gland [117,118]. And BK channels in pituitary tumor (GH3) cells are also activated by ethanol [119]. This potentiation of BK channels in pituitary cells may contribute to the regulation of hormone secretion. Additionally, in a subgroup of primary sensory neurons in rat dorsal root ganglia, the BK potentiation by ethanol reduces neuronal excitability, and therefore, may underlie analgesic action in the peripheral nervous system [120]. Conversely, BK channels in arterial smooth muscle are inhibited by ethanol [121,122]. This ethanol-induced inhibition of BK channels may be a mechanism underlying contraction of vascular smooth muscle. And protracted ethanol exposure decreases ethanol sensitivity and the number of functional BK channels by cellular internalization, resulting in the tolerance to ethanol [118,123].

The response and adaptation of BK channels to ethanol is modulated by the phosphorylation state of the channel protein [124], membrane lipid environment [125–127], and the composition of β auxiliary subunits [123,128]. Moreover, altered expression of splice variants encoding the β core subunit is an underlying mechanism for the ethanol tolerance of BK channels [129]. Pietrzykowski and colleagues reported that, in the supraoptic nucleus and the striatal neurons, brief ethanol exposure decreased the splice variants of BK channels that are highly sensitive to ethanol. This down-regulation of alternatively spliced BK variants is caused by selective degradation of pre-existing mRNA, not by the regulation of alternative splicing. Ethanol exposure increases the expression of a miRNA (miR-9) that binds to 3’UTR sequence specific to the ethanol-sensitive splice variants, and therefore, enhances the degradation of these splice variants. And this molecular tolerance of BK channels to ethanol may contribute to the behavioral adaptation to ethanol.

### 2.6. Neurexin-3

In addition to modulating neurotransmitter receptors and ion channels, ethanol has been reported to inhibit cell–cell adhesion [130] and neurite outgrowth mediated by the L1 cell adhesion molecule (L1CAM) [131]. Ethanol directly binds to L1CAM at a critical domain interface [132] and disrupts its signaling cascade [133], suggesting that cell adhesion molecules are targets of ethanol.

The neuronal proteins known as neurexins function as cell adhesion molecules at presynaptic sites with extracellular binding partners, such as neuroligins, dystroglycan, and neurexophilins [134]. Neurexins modulate postsynaptic differentiation and receptor clustering [135] and perform an essential role in Ca^2+^-triggered neurotransmitter release by modulating voltage-dependent Ca^2+^ channels [136–138]. The gene for one of the neurexin family proteins, neurexin-3, is associated with nicotine dependence [139,140], opioid dependence [141], and poly-substance abuse [142], and cocaine exposure increases neurexin-3 expression in the globus pallidus of mice [143]. Moreover, neurexin-3 is expressed in neurons projecting to brain regions involved in addictive behaviors, such as the nucleus accumbens and the striatum [144] (http://www.brain-map.org/).

Neurexin genes have two distinct promoters from which longer forms (α-neurexins) and shorter forms (β-neurexins) are transcribed. In addition, α-neurexins have five alternative splice sites (splice sites 1–5) and β-neurexins have two (splice sites 4 and 5). In this way, an enormously diverse range of neurexin proteins are generated from each neurexin gene, and this diversity modulates synaptic functions [145]. For example, alternative splicing at splice site 4 modulates the binding affinity and specificity for neuroligins [146].

The splice site producing the greatest variety of proteins is splice site 5 of neurexin-3, which includes three exons (exons 22, 23, and 24). Exon 23 is very important because its inclusion confers solubility on neurexin-3 isoforms, and the transcripts lacking this exon encode transmembrane isoforms. SNP rs8019381, which is in the intronic sequence adjacent to the donor splice site of exon 23, is strongly associated with alcohol dependence and forms a haplotype block with rs760288 and rs2293847 [147]. Furthermore, T allele carriers of rs8019381 express fewer soluble isoforms lacking exon 23. Therefore, this polymorphism might increase the risk of alcoholism by affecting the alternative splicing of exon 23 and modulating the synaptic function of neurexin-3.

## 3. Concluding Remarks

Ethanol consumption can modulate not only the level of total transcripts of multiple genes, but also the ratio of their splice variants. Because splice variants have different functions, the altered expression of splice variants can ultimately affect behavior such as alcohol dependence. Indeed, as mentioned in this review, splice variants of DRD2 may affect ethanol preference by modulating dopamine sensitivity. And lower ethanol-sensitive splice variants of NMDA receptor, GABA_A_ receptor, and ion channels contribute to ethanol tolerance. In addition, splice variants of GABA_A_ receptor, voltage-gated Ca^2+^ channels, and neurexin-3 possibly contribute to withdrawal symptoms by inducing neuronal hyperexcitability. Furthermore, these propensities for ethanol can enhance ethanol consumption in turn. We propose this positive feedback as a mechanism underlying the development of alcoholism. Moreover, among candidate genes mentioned above, DRD2 and neurexin-3 have polymorphisms that are associated with both the risk of alcoholism and the expression ratio of splice variants. Therefore, polymorphisms without amino acid alteration in multiple candidate genes probably increase the risk of alcoholism by modulating the expression of alternatively spliced variants.

Many intriguing questions remain to be answered, such as how ethanol affects the splicing machinery. The oxidative stress induced in ethanol metabolism might be a factor; ethanol consumption results in the production of reactive oxygen species by the mitochondrial electron transport chain, cytochrome P450 2E1, and activated phagocytes [148], and chemical stresses are known to affect the splicing of specific pre-mRNAs [149].

Another question concerns the genome-wide ethanol-associated changes in alternative splicing. Ethanol-associated splice variants have thus far been investigated only for specific genes, not for the entire genome. For a genome-wide study, next generation sequencing is a useful screening tool for detecting both the altered expression of splice variants and alcoholism-associated polymorphisms. And a custom microarray such as the one generated by Johnson and colleagues [150] is also useful for studies of alternative splicing in multifactorial disorders including alcoholism.
